# 

**Published:** 1894-10

**Authors:** 


					﻿Vol XIV.
OCTOBER, 1894.
NO. 10.
THE
JIOMIOPATHIC pHY^lClAfl,
A Monthly Journal of Homoeopathic Materia Medica
and Clinical Medicine.
“ He it a freeman whom truth makes free, and all are slaves betide.”
“Seek the Truth: come whence it may, cot! what it will.”
EDITED AND PUBLISHED BY
WALTER M. JAMES, M. D.
PHILADELPHIA :
ISTo. 1231 LOCUST STREET.
LONDON:
ALFRED HEATH & CO., Sole Agents yob Great Britain,
114 Ebury Street, Eaton Square.
Pearly Subscription, $2.50, invariably in advance. Single numbers, 25 cte.
Inured a* the Philadelphia Peet-Office M Seocad-OleM Mall Matter.
				

